# Notes on the Recent Progress in Ophthalmology

**Published:** 1895-09

**Authors:** Frank C. Todd

**Affiliations:** Fort Worth, Texas


### Current Medical Literature.

### Notes on the Recent Progress in Ophthalmology.

FRANK C. TODD, M. D., FORT WORTH, TEXAS.

STRABISMUS THEORIES.—Hansen-Grist (*Translated by Spalding; Archives of Ophth. XXIV, No. 1.*) In answer to Professor Wahlfors' critique of his theory, Hansen Grist says, that the eyes, when at rest, are in a slightly divergent position, owing to the form of the orbit, the natural length of muscles and the insertion of the optic nerve. This anatomical position of rest is the starting point of every co-ordinate action of the interni. The function the eyes does not allow divergence. Therefore the parallel direction demanded for distance can never be abandoned while awake, and the innervation of convergence is simply maintained by habit. This he calls *unconscious innervation*; or the functional position of rest. He believes that this habit maintains parallelism under the covering hand even with a divergent anatomical position of rest. In some hyphermetropic eyes without binocular vision, in which convergence can not free itself from accommodation, a permanent squint results. And he also thinks that the unconscious habitual innervation, which causes this squint, is maintained even when accommodation is not active.

Convergent squint is the result of an actual innervation of the interni, and divergent squint is a passive process. He denies that one muscle is stronger than the other, but maintains that one is innervated more than the other.

His conclusions are: 1. The convergent position is not anatomical but functional; 2. It is the beginning of the squint, not the cause; 3. The existence of the position previous to the squint can not be proved, but only suspected from experience in certain cases of asthenopia, the significance of which, just at present is not precisely apparent.

---

THE CONSERVATIVE TREATMENT OF MUSCULAR INSUFFICIENCIES.—Heath (*Annals of Ophthal. and Otol., III., No. 2*) gives the following reasons for a moderately conservative treatment of these cases: 1. The eye-muscles are variable in their strength from day to day; 2. Our diagnosis may be wrong. A coincidence for cause and effect, symptoms due to some other cause being attributed to the insufficiency discovered; 3. The difficulty in deciding which is the cause and which the effect in some cases of nervous prostration with insufficiency of one or more eye muscles; 4. Some patients are neurotic by birth and can never be cured. Palliation is all they can expect, and radical treatment becomes hardly justifiable. We should first thoroughly understand the effect of refractive errors and their correction. As errors of refraction frequently cause muscular insufficiencies, correction of the former will often relieve symptoms due to the latter. 6. Regulating the effect of an operation is difficult, if not impossible. It may be too much, too little, or none at all; 7. Results reported by operators are often due to other things than the operation; 8. Some cases show weakness of all the muscles; 9. Simple measures relieve many cases.

---

MICRO-ORGANISMS IN THE CONJUNCTIVAL SAC, AND THE ANTISEPTIC VALUE OF EYE-SALVE.—Bach (*Graefe's Arch. f. Opth , XL., 3*) made some experiments in relation to the presence of bacteria in the conjunctival sac.

The disinfectant action of eye salves commonly in use was tested. The most important conclusions are as follows: 1. The mechanical cleansing of the conjunctival sac with the simultaneous irrigation with physiological salt solution, is preferable to washing the sac with irritating antiseptics. 2. The cleansing of the conjunctival sac, even when normal in appearance, is necessary before an operation. 3. Bandaging the eye acts as a poultice, and favors the growth of germs. All microbes, however, do not find favorable conditions, and the influence of the tears must be considered. 4. Since the cultures from the conjunctival sac of an eye which had been bandaged two days were sometimes sterile, it is proven that the sac can be made sterile. 5. The course of healing depends more upon the technique and the operator, the course of the operation, the patient himself, and the condition of the circulatory apparatus, than upon the quantity of germs in the sac. 6. An infection in the wound should not occur, if proper precautions are taken.

The yellow oxide of mercury salve is somewhat disinfectant, but the bichloride-vaseline salve (1 to 3000), and the 2 per cent. silver and copper salves, even in combination with the tears, are much stronger. They are, in fact, permanently and absolutely sterile. If the sac was not previously specially infected, it is possible to make it sterile, and the margin of the lid, by the use of bichloride-vaselin for twenty-four to forty-eight hours. If numerous colonies of staphylocci are found, the sac will not be rendered sterile, even when the salve has been used eight times in forty-eight hours.

---

THIOFORM IN OCULAR THERAPEUTICS.— Rogman (*Archiv. Oph., XXIV., 2 from Flaudre Médic.*) has obtained favorable results in the treatment of purulent keritits with thioform, used as a substitute for iodoform.

---

DOUBLE SPECTACLES WITH SUPERIMPOSED LENSES, FOR THE USE OF APHAKIC AND HYPEROPIC PATIENTS. — Galezowski (*Rec. d' Oph., 1894*) has invented a frame, with a second clip for the purpose of inserting a second lens in front of the stationary glass. The frequent changing of glasses can thus be dispensed with in any case where a patient is obliged to wear different lenses for near and distant vision.

---

THE PARALLAX TEST FOR HETEROPHORIA.—Duane (*Arch. Oph. Apr., 1895*) gives his reasons for employing the test, and claims for it simplicity, accuracy, and precision. The test is performed in the following manner: The patient is placed at twenty feet from the object of fixation, which is a white spot, one or two *cm.* in diameter, upon a dull black surface of considerable extent. The patient's gaze being directed fixedly at the spot, a card is placed before one eye, and passed alternately from that to the other, the patient being at the same time asked whether the spot appears to move, and if so, what direction. If it remains perfectly stationary, there can have been no deviation behind the card, and the position of fixation of both eyes is perfect. If however, the spot moves, it must occupy a different position as seen by the two eyes; i. e., there is really a diplopia present which our method of observation has unmasked. In order to determine the amount of this alternate diplopia, we place prisms of the appropriate direction and strength before one eye until the movement is abolished.

For near points, the test is made in the same way, a small dot on a rather large card being employed, and the movement of the dot upon the card being observed.

